# Cis-urocanic acid inhibits SAPK/JNK signaling pathway in UV-B exposed human corneal epithelial cells in vitro

**Published:** 2011-08-27

**Authors:** Hanna-Mari Jauhonen, Anu Kauppinen, Tuomas Paimela, Jarmo K. Laihia, Lasse Leino, Antero Salminen, Kai Kaarniranta

**Affiliations:** 1 Department of Ophthalmology, Kuopio University Hospital, Kuopio, Finland; 2 Department of Ophthalmology, Institute of Clinical Medicine, University of Eastern Finland, Kuopio, Finland; 3 BioCis Pharma Ltd, Turku, Finland; 4 Department of Neurology, Institute of Clinical Medicine, University of Eastern Finland, Kuopio, Finland; 5 Department of Neurology, Kuopio University Hospital, Kuopio, Finland

## Abstract

**Purpose:**

The cornea is sensitive to ultraviolet B (UV-B) radiation-induced oxidative stress and inflammation. Its clinical manifestations are photokeratitis and climatic droplet keratopathy. Urocanic acid (UCA) is a major endogenous UV-absorbing chromophore in the epidermis and it is also an efficacious immunosuppressant. We have previously shown that cis-UCA can suppress UV-B-induced interleukin-6 and −8 secretion and cytotoxicity in human corneal epithelium (HCE) cells. In the current study, we further wanted to investigate the effects of cis-UCA on UV-B-induced inflammatory and apoptotic responses in HCE-2 cells, focusing on the nuclear factor kappa B (NF-κB) and AP-1 (subunits c-Fos and c-Jun) signaling pathways.

**Methods:**

After exposing HCE-2 cells to UV-B and cis-UCA, DNA binding of c-Fos, c-Jun and NF-κB was measured with ELISA. In addition, the endogenous levels of phosphorylated stress-activated protein kinase/c-Jun N-terminal kinase (phospho-SAPK/JNK) and phospho-c-Jun were determined. The proliferative capacity of HCE-2 cells was also quantified, and the cytotoxicity of the cis-UCA and UV-B treatments was monitored by measuring the release of lactate dehydrogenase enzyme in the culture medium.

**Results:**

UV-B irradiation induced the binding of transcription factors c-Jun, c-Fos, and NF-κB to DNA. Cis-UCA inhibited the binding of c-Jun and c-Fos but not that of NF-κB. Moreover, UV-B increased the levels of phospho-c-Jun and phospho-JNK, and the expression of both was attenuated by cis-UCA. Cis-UCA also alleviated the UV-B-induced apoptosis and proliferative decline in human corneal cells.

**Conclusions:**

The results from this study suggest that cis-UCA suppresses JNK signaling pathway, which provides potential for treating UV-B-induced inflammatory defects in human corneal cells.

## Introduction

In addition to skin and its epithelial cells, keratinocytes, the eye and its corneal epithelial cells are constantly exposed to ultraviolet (UV) radiation. The acute clinical effect of UV radiation on the cornea is photokeratitis, also known as “snow blindness” or “welder’s flash.” It is a painful inflammatory damage of corneal epithelium caused by UV-B [1,2]. UV radiation accelerates the physiologic loss of surface cells [3,4]. Exfoliation takes place by two mechanisms; shedding where whole cells detach into the tear film and apoptosis in which cells disintegrate into the tear film [1]. Suprathreshold radiant exposure results in full-thickness loss of the stratified epithelium to the basement membrane and, consequently, exposed nerve fiber endings result in severe pain [1].

Climatic droplet keratopathy (CDK) is a degenerative condition characterized by the accumulation of translucent material in the superficial corneal stroma within the interpalpebral strip [5]. The corneal deposits are thought to be derived from plasma proteins which diffuse into cornea, and may become photochemically damaged by excessive exposure to UV [5]. Corneal deposits have been shown to contain various oxidative stress and inflammation–related agents [6-9].

The transcription factors activator protein-1 (AP-1) and nuclear factor-kappaB (NF-κB) are known to be induced by UV-B [10-12]. These two transcription factor families have been identified to be involved in the processes of cell proliferation, cell differentiation and cell survival as well as having important roles in tumorigenesis [12].

The transcription factor NF-κB comprises a family of proteins that are activated in response to inflammatory signals or cellular stress. In NF-κB-dependent gene expression analyses with human keratinocytes, tumor necrosis factor-alpha (TNF-α) and UV-B treatments resulted in the activation and inhibition of different genes, evidence of the stimuli and cell-type specific nature of NF-κB function [13]. NF-κB is activated by direct UV-B exposure and in different pathological conditions of the cornea [14]. During aging, the cellular capacity to respond to environmental stress via NF-κB-mediated signaling can be attenuated [15].

The heterodimeric AP-1 is a transcription factor that is composed of proteins belonging to several families, the Jun (c-Jun, JunB, and JunD) and Fos (c-Fos, FosB, Fra1, and Fra2) subfamilies being the major AP-1 proteins [16]. The AP-1 regulation has been shown to be affected by all forms of mitogen-activated protein kinase (MAPK) cascades, e.g., p38 and JNK (c-Jun N-terminal kinase) [16,17], which activate in response to cellular stress. Study results with human keratinocytes suggest that the activation of p38 MAPK is required for UV-B-induced AP-1 activation. A potential mechanism of UV-B-induced AP-1 activation through p38 is to enhance the binding of the AP-1 complex to its target DNA [18]. Besides p38 activation, a potential UV-B signaling cascade for AP-1 activation in human keratinocytes involves c-Fos gene expression [19,20]. The role of JNK in UV-induced apoptosis is still controversial, studies suggesting either an anti-apoptotic or a pro-apoptotic effect. The biphasic function of JNK can be dependent on cell type, type of stimuli, crosstalk with other signaling pathways, and the intensity and duration of activation [21-23].

UV-B has been shown to induce dose-dependent oxidative stress as well as MAP kinase activation, including JNK, in human corneal epithelium (HCE) cells [10]. In addtion, reactive oxygen species can induce phosphorylation of cell surface receptors, which results in the activation of the MAPK signaling pathway [24]. JNK phosphorylates c-Jun (Ser63/73 and Thr91/93) and potentiates the transcriptional capacity of c-Jun [25-28]. The JNK-initiated phosphorylation of c-Jun has been suggested to increase the half-life of c-Jun by protein stabilization, thus enabling potent and prolonged expression under stressful conditions such as UV irradiation [25,26,29-32]. However, this mechanism seems to depend on the cell type [32,33].

Urocanic acid (UCA) is the major UV-absorbing chromophore in the skin and it has been proposed to function as a regulator of UV-induced damage [34]. Cis-UCA, formed from trans-UCA upon UV-B exposure, has been implicated in the down-regulation of hypersensitivity reactions [35,36], in the actions of epidermal antigen-presenting cells [37,38], in the activation of neutrophils [39,40], and in the prolonged survival of organ transplants [41,42]. Moreover, cis-UCA neither photobinds to DNA [43,44] nor is able to enhance UV photocarcinogenesis [45], whereas it may suppress immunological recognition of tumor antigens in specific experimental conditions [46]. In our previous study, we have showed that cis-UCA suppresses UV-B-induced interleukin (IL)-6 and IL-8 secretion and cytotoxicity in human corneal and conjunctival cells in vitro [47]. However, the molecular targets of cis-UCA action remain to be resolved.

In this study we explored the hypothesis that UV-B radiation causes cell damage through an increase in transcription factor activity and that cis-UCA may protect the exposed corneal epithelial cells through alterations in this activity.

## Methods

### Cell culture

Human corneal epithelial (HCE-2) cells were purchased from American Type Culture Collection (ATCC, Manassas, VA). The cells were cultivated on 6-well cell culture plates (Cellstar^®^, Greiner Bio-One, Frickenhausen, Germany) in Keratinocyte-SFM medium (Gibco, Invitrogen, Paisley, UK) supplemented with 50 µg/ml bovine pituitary extract, 5 ng/ml human recombinant epidermal growth factor, 100 U/ml penicillin, 100 µg/ml streptomycin (all from Gibco), 5 µg/ml insulin (Sigma-Aldrich, St. Louis, MO), and 10% fetal bovine serum (HyClone, Logan, UT). Confluent cell cultures were treated with 100 µg/ml of cis-UCA (BioCis Pharma, Turku, Finland) when indicated in Results, and/or exposed to a UV-B irradiation dose of 153 mJ/cm^2^ (four TL 20W/12 tubes; Philips, Eindhoven, The Netherlands) at room temperature for 1 min using a source-to-target distance of 30 cm. Thereafter, the cell cultures were incubated in a humidified 10% CO_2_ incubator at 37 °C for 3, 6, or 24 h.

### ELISA assays

For determining the DNA binding of transcription factors and for analyzing the activation of AP-1, cell lysates were prepared by scraping the cells into Buffer C (25% glycerol, 0.42 M NaCl, 1.5 mM MgCl_2_, 0.2 mM EDTA, 20 mM Hepes in double-distilled water). To detect the binding of AP-1 and NF-κB to DNA, c-Fos and c-Jun TransAM^™^ kits (Active Motif, Rixensart, Belgium), and NF-κB p65 ELISA Kit (Enzo Life Sciences, Farmingdale, NY) were used. Phosphorylated c-Jun and phosphorylated stress-activated protein kinase/Jun-N-terminal kinase were measured using PathScan^®^ Phospho-c-Jun (Ser63), and PathScan^®^ Phospho-SAPK/JNK (Thr183/Tyr185) Sandwich ELISA Kits (Cell Signaling Technology, MA), respectively. All assays were performed according to the manufacturers’ protocols. The absorbance values were measured at 450 nm with a reference wavelength of 655 nm using a BIO-RAD Model 550 microplate reader (BIO-RAD, Hercules, CA).

### Proliferation assay

For the proliferation test, 100,000 cells/ml were plated in 200 µl on 96-well flat-bottomed cell culture plates (Cellstar^®^, Greiner Bio-One). After 3 h of incubation in a humidified 10% CO_2_ incubator at 37 °C, cells in eight replicate wells were treated with cis-UCA and UV-B irradiation as described above. The cell cultures were incubated in a humidified 10% CO_2_ incubator at 37 °C for 24 or 48 h, and the proliferation of HCE-2 cells was quantified using the CyQUANT^®^ Cell Proliferation Assay Kit (Invitrogen) according to the manufacturer’s instructions. After 2 min incubation at room temperature, fluorescence intensity of the samples was measured at the ex/em wavelength of 485/530 nm using VICTOR^™^ 1420 multilabel counter (PerkinElmer/Wallac, Turku, Finland).

### Cytotoxicity assay

Cytotoxicity of the cis-UCA and UV-B treatments was monitored by measuring the amount of lactate dehydrogenase (LDH) enzyme in duplicate from the culture medium samples. Cyto-Tox 96 Non-Radioactive Cytotoxicity Assay kit (Promega, Madison, WI) was used for detection according to the instructions of the manufacturer. Absorbance values after the colorimetric reaction were measured at the wavelength of 490 nm with a reference wavelength of 655 nm using a BIO-RAD Model 550 microplate reader (BIO-RAD).

### Statistical analysis

Statistical differences between groups were assessed using the Kruskall-Wallis test, and post hoc comparisons were made using the Mann–Whitney U-test. P values below 0.05 were considered significant.

## Results

**Figure 1:**
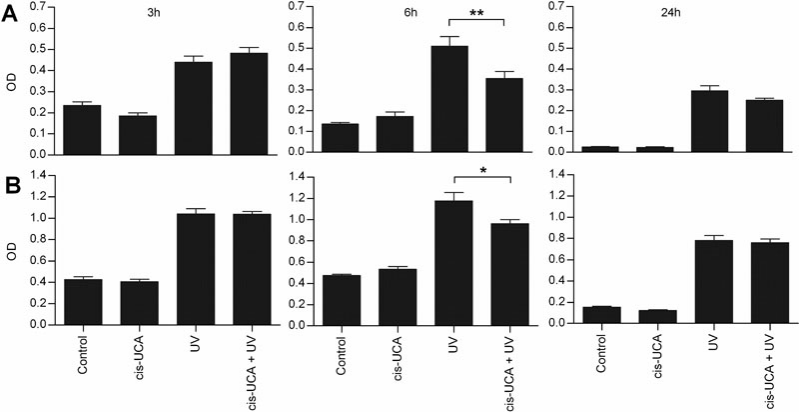
DNA binding of c-Fos and c-Jun subunits of the transcription factor AP-1 heterodimer. Binding of c-Fos (**A**) and c-Jun (**B**) to DNA. Results are presented as mean optical density (OD) ± SEM cis-UCA concentration was 100 µg/ml. Seven parallel samples were measured in control and cis-UCA, and nine parallel samples in UV and cis-UCA + UV treatments. *p<0.05; **p<0.01 (Mann–Whitney).

**Figure 2:**
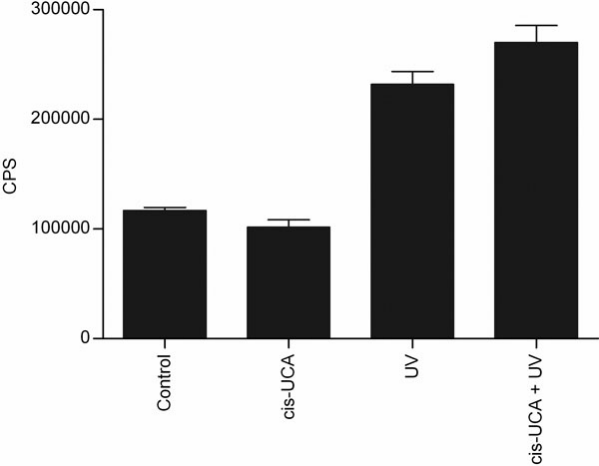
Binding of NF-κB (p65) to DNA 6 h after stimulation. Results are presented as mean counts per second (CPS) ±SEM cis-UCA concentration was 100 µg/ml. Five parallel samples were measured in control and cis-UCA, and seven parallel samples in UV and cis-UCA + UV treatments.

To examine the activity of central transcription factors following UV-B and cis-UCA treatments, the DNA binding of AP-1 and NF-κB were determined. The DNA binding of c-Fos and c-Jun subunits of the transcription factor AP-1 heterodimer increased following UV-B irradiation (Figure 1). After 6 h of incubation, cis-UCA significantly decreased the UV-B-induced binding of both subunits (Figure 1). The decrease was not yet observed at 3 h of incubation, and it was negligible after 24 h. UV-B irradiation also approximately doubled the binding activity of the p65 subunit of NF-κB when compared to non-irradiated control cells after 6 h of incubation. However, cis-UCA did not affect this activation at any of the 3, 6 or 24 h time points studied (Figure 2 and data not shown). No significant change in the activity of AP-1 or NF-κB was observed in non-irradiated cells treated with cis-UCA (Figure 1 and Figure 2).

**Figure 3:**
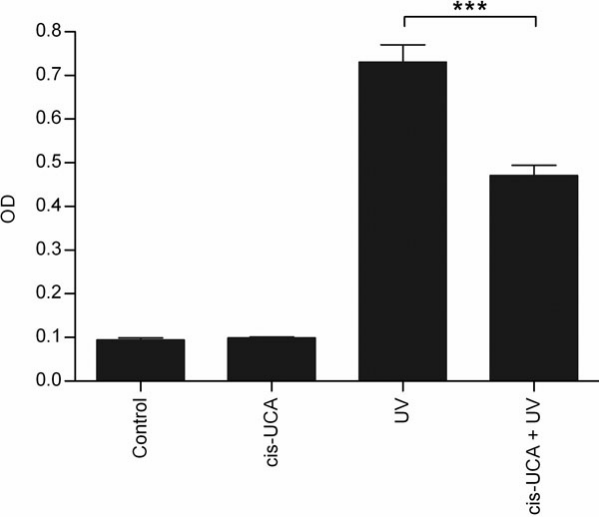
Phosphorylation of c-Jun (Ser63) 6 h after stimulation. Results are presented as mean optical density (OD) ±SEM cis-UCA concentration was 100 µg/ml. Seven parallel samples were measured in control and cis-UCA, and nine parallel samples in UV and cis-UCA + UV treatments. ***p<0.001 (Mann–Whitney).

**Figure 4:**
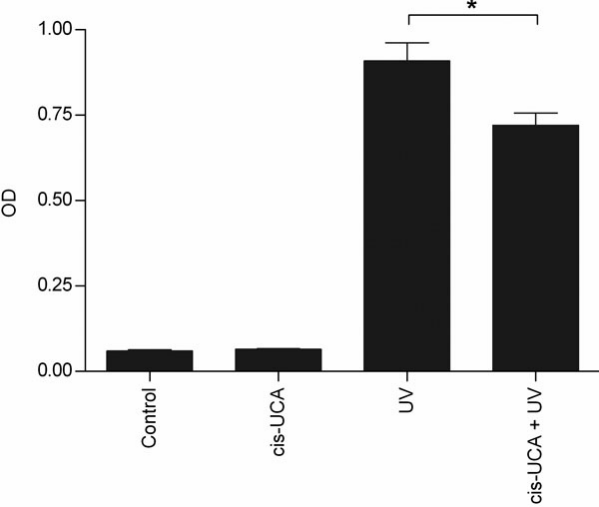
Phosphorylation of SAPK/JNK (Thr183/Tyr185) 6 h after stimulation. Results are presented as mean optical density (OD) ±SEM cis-UCA concentration was 100 µg/ml. Seven parallel samples were measured in control and cis-UCA, and nine parallel samples in UV and cis-UCA + UV treatments. *p<0.05 (Mann–Whitney).

To verify our observation that cis-UCA inhibits the activity of AP-1 in UV-B-irradiated HCE-2 cells, we measured phosphorylated c-Jun from the cell extracts. As shown in Figure 3, cis-UCA significantly decreased the level of phospho-c-Jun in UV-B-irradiated HCE-2 cells after 6 h of incubation. Moreover, cis-UCA also significantly decreased the amount of phosphorylated JNK in those cells (Figure 4). In non-irradiated cells cis-UCA had no effect on the expression of these phosphoproteins.

**Figure 5:**
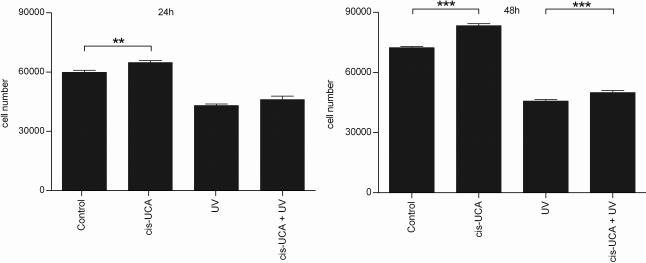
Proliferation of HCE-2 cells. Results are presented as mean cell numbers ±SEM cis-UCA concentration was 100 µg/ml. Eight parallel samples were measured in all groups. **p<0.01; ***p<0.001 (Mann–Whitney).

**Figure 6:**
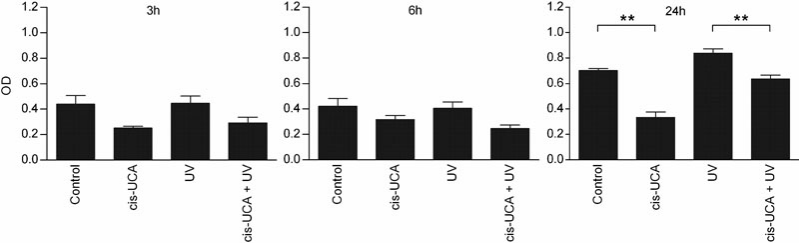
Release of lactate dehydrogenase (LDH). Results are presented as mean optical density (OD) ±SEM cis-UCA concentration was 100 µg/ml. Six parallel samples were measured in all groups. **p<0.01 (Mann–Whitney).

Since JNK signaling can result in either cellular proliferation or apoptosis [48], we examined the influence of cis-UCA on cell survival. As shown in Figure 5, cis-UCA significantly prevented the loss of viability of HCE-2 cells in normal cell culture conditions. Also after UV-B irradiation, cell survival was increased by cis-UCA after 24 h of incubation and reached statistical significance after 48 h (Figure 5). Concomitantly, cis-UCA decreased cell damage. The decreased release of LDH from cis-UCA-treated cells was observed at 3 and 6 h of incubation and was statistically significant after 24 h both in non-irradiated and in UV-B-exposed HCE-2 cells (Figure 6).

## Discussion

We have previously shown that cis-UCA suppresses UV-B-induced cytokine expression and improves cell viability against UV-B irradiation in human ocular cells [47]. In the present study, we further investigated the mechanisms of these actions. As the cornea is frequently exposed to solar UV radiation, we wanted to elucidate the role of JNK in apoptotic regulation in HCE-2 cells. Our data demonstrates that cis-UCA inhibits the phosphorylation of c-Jun (Ser63) and JNK (Thr183/Tyr185) as well as the binding of c-Fos and c-Jun to DNA in response to UV-B stimulation. The findings that cis-UCA reduced the phosphorylation of both JNK and c-Jun, and had no effect on basal level of these phosphoproteins in non-irradiated cells, suggests that the molecular target of cis-UCA action is up-stream of JNK in UVB-stressed cells.

UV-induced generation of reactive oxygen species and subsequent TNF-α formation activates, besides JNK signaling, also the NF-κB pathway [49,50]. JNK activation by TNF- α activates pro-apoptotic effects; however, TNF-α-induced NF-κB activation prevents apoptosis through the suppression of the JNK pathway and the activation of antioxidant genes, such as manganese-superoxide dismutase (MnSOD) [49,50]. Interestingly, in epidermal cells, JNK activates cell proliferation, and the inhibition of JNK by NF-κB has a tumor-suppressing function [51].

Conversely, following UV stimulus, p65/RelA directly results in the expression of protein kinase C delta (PKCδ), which leads to activation of JNK [52]. In addition, after UV stimulation, MnSOD expression has been shown to be UV dose-dependent, exerting diminishing expression in high UV-B doses [10]. However, at the same time, UV-B exposure induces the NF-κB-related proinflammatory cytokines IL-6 and IL-8 in HCE-2 cells [47]. Our research shows that both JNK and NF-κB pathways are activated by UV-B. However, cis-UCA suppresses solely the JNK pathway, not NF-κB. In response to UV-B stress, HCE-2 cells showed decreased proliferation and increased LDH release, implying cell death, which could be alleviated by cis-UCA. Consistently with an earlier study with epidermal cells [51], the inhibition of JNK pathway seems to be a critical target in the regulation of apoptosis also in human corneal epithelial cells. While cis-UCA was present in the culture medium during UV-B irradiation of the cells, it was inferred from previous experience [47] that the cis-UCA concentration used in the current experiment does not appreciably block the transmission of UV-B photons.

Although acute and chronic damage and inflammation caused by UV radiation to the epithelial cells of the cornea are well known ophthalmologic diseases (e.g., photokeratitis and CDK), their precise mechanisms are still unclear. Current clinical therapy for ocular surface inflammation consists of anti-inflammatory agents that do not offer any protection against UV radiation-induced damage [53]. The present in vitro data show that cis-UCA regulates the JNK signaling pathway and it has at both anti-inflammatory and cytoprotective capacity against UV radiation on the corneal epithelial cells. Our results are supported by previous observations [54]. cis-UCA may be useful also in other inflammatory conditions of the cornea [55,56]. It would be worthwhile to examine cis-UCA effect on these diseases as well. Therefore, further in vivo studies are required.
